# Diagnostic and treatment challenge in adult presentation of congenital pseudoarthrosis of the tibia: A case report

**DOI:** 10.1016/j.amsu.2020.08.030

**Published:** 2020-08-29

**Authors:** Faisal Miraj, Dina Aprilya

**Affiliations:** Department of Orthopaedic and Traumatology, Faculty of Medicine Universitas Indonesia, Fatmawati Hospital, Indonesia

**Keywords:** Adult congenital pseudoarthrosis, Ilizarov method, Misdiagnosis

## Abstract

**Introduction:**

Congenital pseudoarthrosis of the tibia is a rare congenital disease. Late presentation in adult, makes the diagnostic far more challenging and often misdiagnosed as a common non-union fracture with high re-operation rate. In long courses of repeated surgery, non-union persisted along with severe leg length discrepancy.

**Case presentation:**

A 19-year-old male presented with history of left tibia fracture with repeated surgery. Current problems were progressed bowing of the left lower leg and length discrepancy without recent injury. There was a sign of neurofibroma and pseudoarthrosis at distal third of the tibia shaft with fibula involvement. A radical resection was performed followed by staged deformity correction with Ilizarov's method which was consisted of bone transport procedure in 4 months and lengthening procedure in another 4 months, without grafts.

**Discussion:**

After one-year, patient achieved union at docking site, equal lower limb length, good alignment and consolidation in bone transport and lengthening site. Three months after frame removal patient has achieved functional bipedal gait with no sign of recurrence.

**Conclusion:**

Diagnostic and therapeutic challenge in the late onset of congenital pseudoarthrosis of tibia is in differentiating it with another cause of non-union and in dealing with deformities. Although none of surgical methods have proven their superiority, reconstruction using Ilizarov method is proved to be safe, practical, and effective to solve both problems. However, the patient still needs to be closely observed and protected weight bearing due to the refractory nature of the disease.

## Highlights

• No gold standard of treatment in congenital pseudoarthrosis.
• Late presentation of CPT makes the diagnostic far more challenging and it often misdiagnosed as a common non-union fracture with high re-operation rate.
• Ilizarov method is not a modern technique but it is proved to be safe, simple, practical, and effective to solve both deformity and pseudoarthrosis problems in CPT.

## 1 Introduction

Congenital pseudoarthrosis of the tibia (CPT) is a rare congenital condition and usually presents in early childhood. If present late in adolescent or adult, the condition will be more challenging in differentiating with another etiology as well as managing more advance problems such as severe deformity, leg length discrepancy (LLD), long-standing contracture and disuse atrophy or osteopenia in associated limb. Different options have been proposed to overcome the deformity and CPT's refractory nature. However, outcomes are varying and often unsatisfactory [1,2].

We presented a case of 19-year-old male with an adult presentation of CPT. Staged surgeries were performed consisted of pathological segment resection, bone transport and lengthening with Ilizarov method. Clinico-radiographical outcomes were reported [3]. Written consent has been received from the subject. The authors declare no conflicts of interest. This work has been reported in line with the SCARE criteria [4].

## 2 Case presentation

A 19-year-old male presented with a recurrent angular deformity and severe LLD of left lower leg after several surgeries in other hospitals due to tibia fracture. The first fracture was occurred when he was 13-year-old caused by falling from the chair. There was no sign of union after initially treated by bone setter for 6 months. Then, patient was brought to orthopedic surgeon and treated with open reduction internal fixation (ORIF) with plate and screw. Four years later, the patient had undergone several revision ORIF because his bone was re-fractured with trivial mechanism in 8–12 months interval. Lastly, the implant was removed, and the bone was debrided with suspicion of infected non-union. However, tissue culture confirmed no infection. No family history with the similar condition. The patient was then referred to Fatmawati General Hospital, Jakarta, Indonesia, for further diagnostic work-up and treatment.

**Fig. 1:**
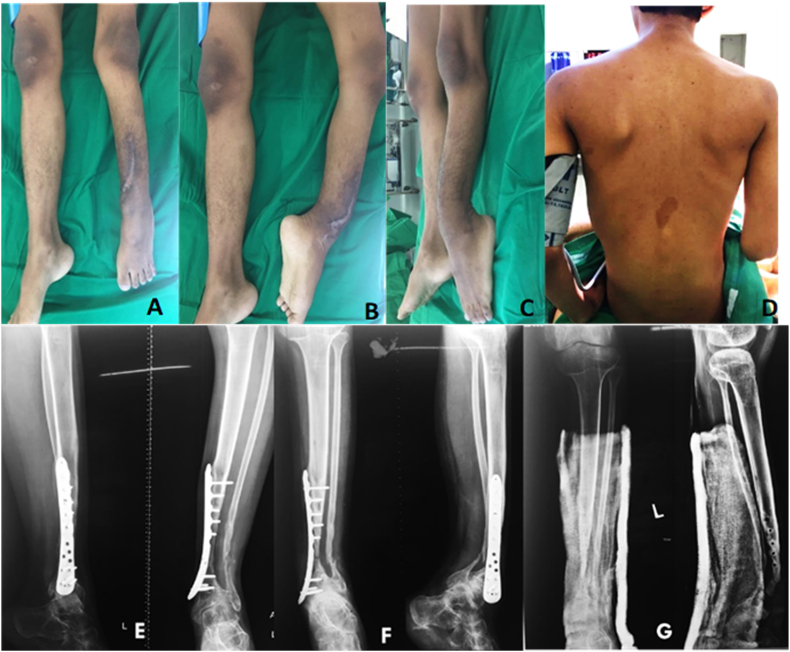
Clinical picture at first admission. **A-C** Antero-lateral angulation and shortening. No signs of infection from the previous scar and surroundings. **D.** Café au lait spots on patient's back. **E.** Third revision surgery. **F.** Failure 8 months after third revision: Loosening of the distal screws and anterolateral angulation with bone discontinuity. **G.** Plain radiograph after implant removal: Tapering of both fracture ends.

**Fig. 2:**
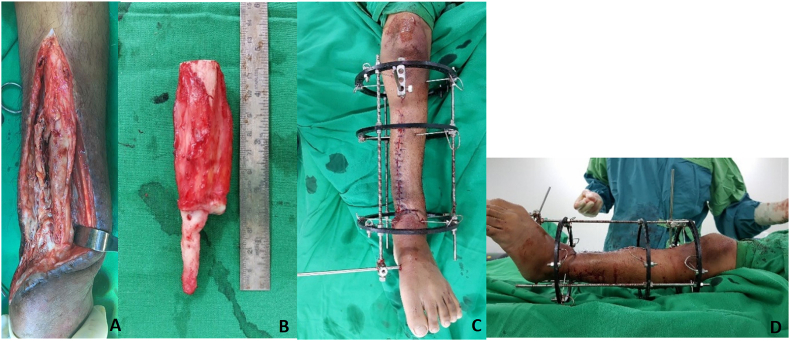
Intraoperative procedure. **A.** Dystrophic segment of tibia and fibula from middle to distal third of tibia and fibula, no signs of infection. **B.** Pathological fibrous tissue at the pseudoarthrosis site and on the surrounding periosteum was resected. **C-D** Final Ilizarov's construct for bone transport after angulation deformity correction and maintain the bone and joint axis.

At the physical examination there was bowing of left lower leg with 10 cm LLD. There were no soft tissue defect, dehiscence, or sinuses observed at the previous scar and surroundings (Fig. 1A–C). Café au lait spots were found at the back with varying sizes from 3 mm to a large macule of 15 × 25 mm (Fig. 1D). From serial radiograph, both fracture ends were gradually tapered from time to time. Bone discontinuity was persistent, and the implant failed to fixed the fragment in physiologic loading (Fig. 2E and F). After implant removal, cortical thickening which reduced the medullary canal were seen on both tapered end (Fig. 2G).

Surgery was performed by 1 senior author (F.M.) who is experienced in the field of pediatric orthopedic and limb deformity correction in a single academic orthopedic surgical practice. At the first stage, a meticulous resection of sclerotic bone edges and surrounding fibrous hamartoma was performed at the junction of middle and distal segment of tibia and fibula as well (Fig. 2A and B). After that, the angular deformity was corrected. The limb alignment and joint orientation were restored and maintained. Stable construction was achieved by Ilizarov's frame consisted of three circular frames coupled with transfixing wires and pins located at proximal, middle, and distal segment of tibia (Fig. 2C and D). Osteotomy was performed at proximal segment between proximal and middle ring to allow bone transport in order to fill 12 cm defect after resection. Intramedullary pin was utilized to guide bone transport process. The pathological result from the resected pseudoarthrosis showed a mature lamellar bone tissue surrounded with soft tissue consisted of spindle cell proliferation.

**Fig. 3:**
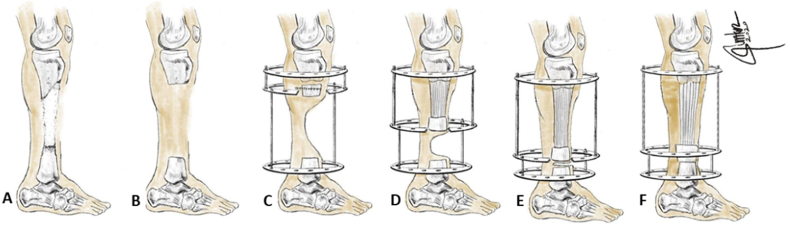
Illustration of Ilizarov Method. **A**. Pseudoarthrosis over the middle to distal third tibia. **B.** After resection, leaving a critical bone defect. **C.** Proximal segment osteotomy for bone transport. **D.** Bone transport process. **E.** Folded skin at docking site. **F.** Docking site reconstruction to facilitate docking with fresh and viable bone edges, followed by lengthening process.

Bone transport performed after a week with 1 mm daily divided into four times. At the fourth months of transport process, transported bone segment which almost reached docking site with 10 cm length of transport was interfered with skin invagination at docking site. Next surgery was performed to remove the embedded soft tissue, compress the docking site, and readjust the axis. Intraoperatively, both edges of contact point were viable for union. Therefore, bone grafts were unnecessary. The tibia was further lengthened while the docking site was compressed between middle and distal ring, until it reached the same length as the opposite side in four months more, which was reached 10 cm. The frame was maintained until callus consolidation at one year. Intravenous bisphosphonate administration was given annually to improve bone healing biologically. The summary of method is illustrated in Fig. 3.

**Fig. 4:**
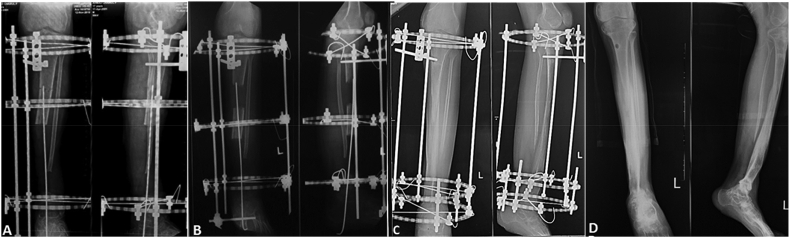
Serial radiological examination (A–D)**. A.** After wide resection and ilizarov frame application. **B.** Two months after surgery, bone transport process. **C.** At one year after surgery, union docking site and consolidation of transport and lengthening site. **D**. At 6 months follow-up after frame removal, solid union achieved at both transport and docking site.

**Fig. 5:**
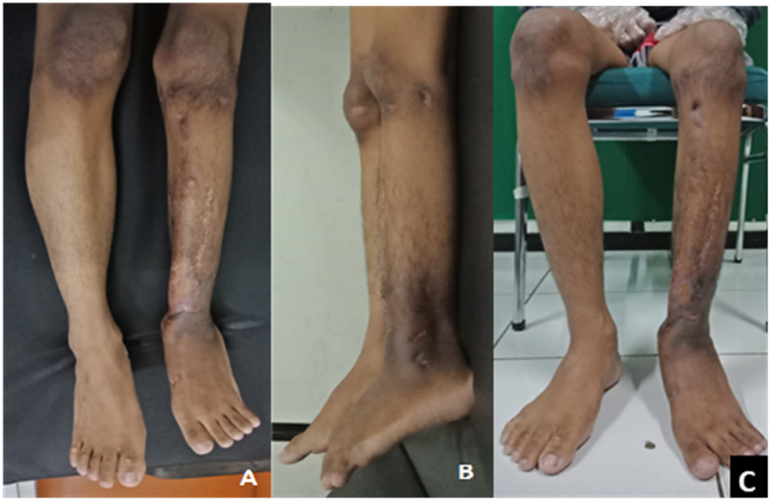
Clinical appearance at the latest follow-up. **A-C.** Equal limb length had been achieved with acceptable plantigrade ankle position and well-healed wound.

Serial radiological examinations were done (Fig. 4). At one-year follow-up, the limb length was restored, and radiological examination showed union at docking site and callus consolidation at transport and lengthening site. The frame was removed, and the patient started to practice partial weight bearing. At another 6 months, a solid union had achieved. The wound had healed nicely, no significant bowing and length discrepancy and patient had walk with protected weight bearing (Fig. 5).

## 3 Discussion

CPT is a rare disease characterized by segmental osseous weakness with pseudoarthrosis resulting in anterolateral angulation and subsequently proceed to fragility fractures which hardly achieved bony union. The incidence is between 1:140,000 to 1:250,000 neonates. Although majority of the disease apparent within the first year of life, Andersen described a rare late onset CPT type which develops between the ages of 4 and 12 years. The etiology remains unknown. Up to 50% of the cases is associated with neurofibromatosis type I (NF-1) and with lesser extent is associated with fibrous dysplasia [[5], [6], [7], [8]].

This patient had several trivial injuries preceding to fracture with no previous history of musculoskeletal abnormality that started by the age of 13. Patient had undergone five times surgeries to overcome those fractures. This repeated surgery might be caused by failure of recognition of the evolving CPT in such a late presentation. Therefore, pathological tissue resection was inadequate besides the nature of the CPT itself that caused poor healing process.

In this case, other possibility of mechanical and biological condition must be elaborated to plan the management accordingly. Infection has been excluded. No smoking habits and other nutritional factors which may interfere with bone healing. In this patient, NF-I was suspected based on cafe-au-lait macules with the size of more than 15 mm, pathological finding and it was more-likely to be the base pathology and linked to the pseudoarthrosis condition [3,[9], [10], [11]].

According to El-Rosassy-Paley classification, this patient had CPT type 2 with an antero-lateral bowing and a mobile pseudoarthrosis of the bone that have been through multiple failed surgeries. Thus, biologic enhancement of bone healing and managing the remaining bone defect either by bone transport or lengthening procedure are suggested [3,8]. Surgical is mainstay treatment to achieve union, restore alignment, prevent recurrence and limit LLD while preserving articular function. Although many methods have been proposed, the superiority of one treatment over another remains difficult to determine. The resection of the fibrous hamartoma and of the associated diseased periosteum plays a role in preventing recurrence [3]. Next, both mechanical and biological problem must come into consideration to achieve successful bony union. Various studies proposed many kinds of fixation devices such as external fixators, intramedullary fixation, and plate and screw. Biological factors can also utilized such as bone graft, periosteal graft, osteo-inductive proteins and bisphosphonates [2,3,7,8].

The Ilizarov method was firstly introduced during late 1980s to treat bone defects and also has been popularized to the treatment of CPT. The biological concepts behind this method are removing the pathological tissue, recreating new healthy environment and aiming to fill the defect by distraction osteogenesis concept through bone transport procedure while correcting the deformity simultaneously. The mechanical stability that is mandatory for optimization of bone healing, is accomplished through the ring frame supports and the use of transfixion wires and half pins. Furthermore, this frame is cheaper than other modality and in combination with shorter hospitalization length made this method is economically valuable. Recent study of 12 patients treated with a bone transport reported excellent results in eight patients, good in three patients and poor in one patient. Other study showed union in 22 out of 23 patients, with a mean follow-up of 9.2 years (four bone transports) [12,13].

Despite the method used, the refracture is not uncommon. Association of NF to the final outcome is still debatable. Other factors such as age at treatment less than three years, previous failed surgery and years of follow-up also were reported to negatively affect union [1,8,[12], [13], [14], [15], [16]]. The patient had reached skeletal maturity when treated with Ilizarov method. This is a positive prognostic factor for bone healing in this case. The patient also had a good compliance and satisfied with this method of treatment which is proven to be safe and effectively overcome the deformity with good bone healing improvement as seen at follow-up radiographs. At the 18 months follow-up, patient had developed bony union with full weight bearing and normal gait (Johnston's stage 1).

This study has some limitations. Although the medium-term result is satisfactory, long term follow-up must be done and the use of protective brace is still advocated to prevent re-fracture and deformity recurrence. The number of cases also needed to be increased to support the evidence using this method.

## 4 Conclusion

In the late presentation of congenital pseudoarthrosis of tibia, diagnosis is challenging since surgeon must exclude many possibilities of non-union fracture. Surgeon may also face additional problem such as severe deformity. No gold standard among many proposed treatments and outcomes are varies.

The Ilizarov method is a safe, simple, effective, and practical option for patients with congenital pseudoarthrosis of tibia. It can achieve several goals such as union of the pseudoarthrosis, correct deformity as well as leg-length equalization. However**,** re-fracture is the main issue since the bone often heals with a poor biological or mechanical quality. Life-time protections with orthosis, walking aid and fall prevention are advisable.

## Consent for publication

Written informed consent was obtained from the patient for publication of this case report and accompanying images. A copy of the written consent is available for review by the Editor-in-Chief of this journal on request.

## Funding

This research did not receive any specific grant from funding agencies in the public, commercial, or not-for-profit sectors.

## Provenance and peer review

Not commissioned externally peer reviewed.

## Guarantor

Faisal Miraj is the sole guarantor of this submitted article.

## Author contribution

Faisal Miraj contributes in the study concept or design, data collection, analysis and interpretation, oversight and leadership responsibility for the research activity planning and execution, including mentorship external to the core team

Dina Aprilya contributes to the study concept or design, data collection, analysis and interpretation, and writing the paper.

## Ethical approval

Ethical approval was not required in the treatment of the patient in this report.

## Disclaimer

No patient or author details are included in the figures.

## Declaration of competing interest

None.
